# Recovery of strain-hardening rate in Ni-Si alloys

**DOI:** 10.1038/srep15532

**Published:** 2015-10-21

**Authors:** C. L. Yang, Z. J. Zhang, T. Cai, P. Zhang, Z. F. Zhang

**Affiliations:** 1 Shenyang National Laboratory for Materials Science, Institute of Metal Research, Chinese Academy of Sciences, 72 Wenhua Road, Shenyang 110016, P.R. China

## Abstract

In this study, the recovery of strain-hardening rate (RSHR) was discovered for the first time in polycrystalline materials (Ni-Si alloys) that have only dislocation activities during tensile test. Detailed microstructure characterizations show that the activation of dislocations in the secondary slip systems during tensile deformation is the major reason for this RSHR. By taking into account other metals that also exhibit RSHR during tension, a more general mechanism for the RSHR was proposed, i.e. the occurrence of a sharp decrease of dislocation mean free path (Λ) during plastic deformation, caused by either planar defects or linear defects.

As fundamental mechanical properties, strength and ductility determine the applications of structural materials^1^. However, a general strengthening process usually results in the reduction of plasticity, which was often referred to as the so-called trade-off relationship between strength and ductility^2^,^3^,^4^. To overcome this trade-off relationship, several approaches were developed to synchronously improve the strength and plasticity of materials, such as, introducing high density of nano-twins^5^,^6^,^7^, adjusting dislocation slip mode through alloying^8^ and manufacturing gradient microstructure through surface mechanical treatment^9^,^10^,^11^. Intrinsically, all these methods follow the same basic mechanism, to improve the strain hardening capacity^10^,^12^,^13^. According to the considére criterion^14^, during the tensile process of ductile metals, the strain-hardening rate (Θ) decreases monotonously with the increase of strain until its value equals to the true stress, and then necking happens. Therefore, the recovery of strain-hardening rate (RSHR) is significantly important for the simultaneous improvement of strength and ductility.

Up to now, the RSHR can be only achieved in two kinds of metals, twinning induced plasticity (TWIP)^15^,^16^,^17^ and transformation induced plasticity (TRIP) alloys^18^,^19^, both of which truly exhibit high strength and large plasticity. The basic mechanism for the RSHR of these alloys is that defects other than dislocations (twin boundaries for TWIP alloys and phase boundaries for TRIP alloys) appear during plastic deformation, which brings dynamic Hall-Petch effect^20^,^21^,^22^,^23^. Thus, it seems impossible to realize the RHSR in those traditional polycrystalline materials whose microstructure only consists of dislocations during plastic deformation^24^. Moreover, theoretical calculations based dislocation theories also reveal that Θ at most remains constant with the increase of strain for the materials with dislocation slip alone^24^,^25^. Nevertheless, if considering the case of single crystals, we will find that this RSHR effect also arises obviously when the single crystals are oriented for single slip (from strain-hardening stage I to II). Thus, this gives rise to two open questions: **(1) whether the polycrystalline metals with only dislocation slip mechanism may possess RSHR effect? and (2) what is the general mechanism for the RSHR?**

As the decrease of Θ during deformation is mainly driven by the annihilation of screw dislocations at room temperate, which is facilitated by dislocation cross slip^8^, we should select the materials in which dislocations glide in planar mode, in order to get possible RSHR. On the other hand, deformation twins, stacking faults or second phases should be deliberately avoided during plastic deformation, so the materials with extremely low stacking fault energy (SFE) or phase transformation must be excluded. In view of these restrictions, we chose a novel single phase solid solution with medium SFE, Ni-Si alloys, in the present study to carry out tensile tests and relevant investigations, aiming at seeking for the RSHR materials other than the TWIP or TRIP alloys and further revealing the general and intrinsic mechanism for the RSHR effect.

## Results

### Basic mechanical properties of Ni-Si alloys

**Figure 1:**
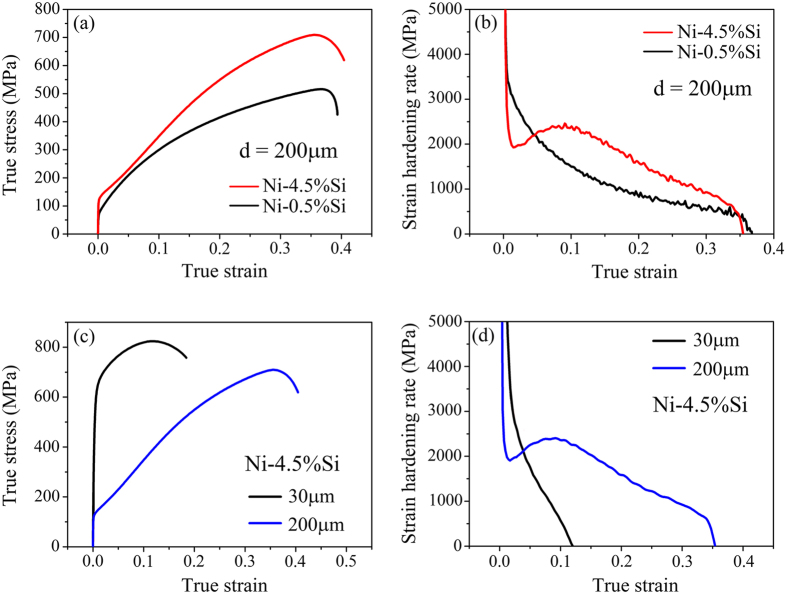
True stress-strain and strain-hardening rate curves of Ni-0.5 wt.%Si and Ni-4.5 wt.%Si with different grain sizes. It can be seen that only Ni-4.5 wt.%Si with large grain size (200 μm) exhibits the RSHR effect.

Figure 1 shows the results of tensile tests, including stress-strain curves and strain-hardening rate curves. Figure 1(a) exhibits the true stress-strain curves of Ni-4.5 wt.%Si and Ni-0.5 wt.%Si alloys annealed at 1000°C (with grain size of ~200 μm). Obviously, the ultimate tensile strength of Ni-4.5 wt.%Si alloy is higher than that of Ni-0.5 wt.%Si alloy for about 200 MPa. Meanwhile, the ductility of these two alloys are nearly the same. Noticing that the difference in the yield strength between the two alloys is relatively small, such a promotion in the ultimate tensile strength can be only contributed to the improved strain hardening capacity of Ni-4.5 wt.%Si alloy. Figure 1(b) shows the strain-hardening rate curves of the two alloys, from which it is interesting to find that the Θ of Ni-4.5 wt.%Si alloy recovers obviously (about 500 MPa), while it decreases monotonously for Ni-0.5 wt.%Si alloy during tension. This obvious RSHR in the polycrystalline Ni-4.5 wt.%Si alloy has never been observed in other polycrystalline metals except for the above mentioned TWIP or TRIP alloys^16^,^26^,^27^. This indicates that the Ni-Si alloy component is one key factor for this RSHR. In order to further examine the effect of grain size, we prepared another microstructure of Ni-4.5 wt.%Si alloy with a relatively smaller grain size and carried out tensile tests. Figure 1(c) displays the tensile stress-strain curves of the Ni-4.5 wt.%Si alloy with different grain sizes, and Fig. 1(d) depicts the corresponding strain-hardening curves. From Fig. 1(d), it is obvious that the RSHR phenomenon only arises in Ni-4.5 wt.%Si alloy with coarse grain (200 μm). Besides, as the grain size decreases, Θ reduces quickly with the increase of strain. Therefore, the appearance of RSHR should be not only affected by alloy component, but also by the microstructure, i.e. grain size.

### Typical microstructures of Ni-Si alloys after tension

**Figure 2:**
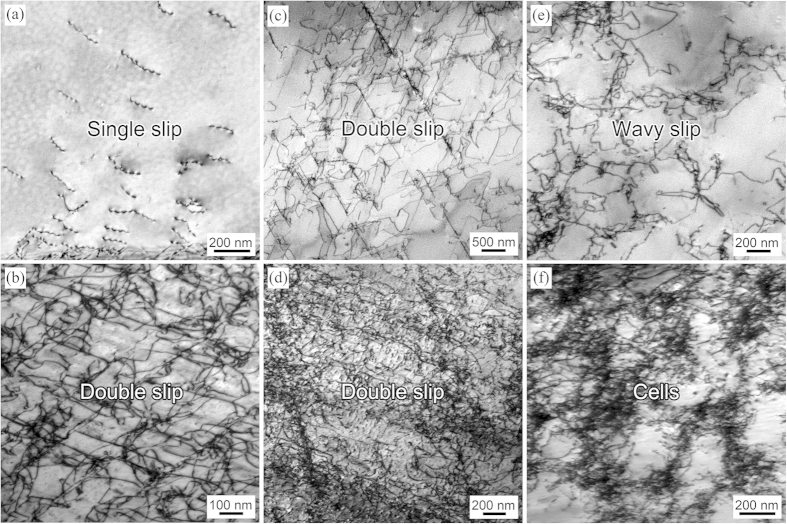
Typical microstructures of Ni-4.5 wt.%Si with grain sizes of 200 μm (**a,b**) and 30 μm (**c,d**), and Ni-0.5 wt.%Si of 200 μm (**e,f**) at different true strain (0.02 for (**a,c,e**) and 0.13 for (**b,d,f**)). Only dislocation morphologies were observed in all samples after tension. The transformation from single slip to double slip during deformation in Ni-4.5 wt.%Si with large grain size is the key cause for RSHR.

Figure 2 shows the microstructures of Ni-4.5 wt.%Si and Ni-0.5 wt.%Si alloys with different grain sizes. It can be seen that there are only dislocation structures in morphology for all the samples with different grain size and tensile strain, while deformation twins, stacking faults or second phases cannot be detected. Overall, Ni-4.5 wt.%Si alloy exhibits planar slip characteristic in either large ((a) and (b)) or small grain sizes ((c) and (d)); while Ni-0.5 wt.%Si alloy displays wavy slip feature ((e) and (f)) that is similar to pure Ni during plastic deformation^28^. With the increase of strain, dislocation density increases obviously for all the three materials, however, both the slip mode and grain size affect the dislocation multiplication processes greatly. For Ni-4.5 wt.%Si alloy with a grain size of 200 μm, only single slip system was detected in most grains at a small strain of 0.02 just before the RSHR (Fig. 2(a)), and double or multi slip systems were triggered with the increase of strain (Fig. 2(b)). Nevertheless, for Ni-4.5 wt.%Si alloy with smaller grain size (30 μm), double slip systems were activated from the very beginning of plastic deformation (Fig. 2(c)) and further deformation only increases the dislocation density (Fig. 2(d)). For Ni-0.5 wt.%Si alloy with wavy slip mode, tangling dislocations were found at a small strain (Fig. 2(e)), which finally evolved into dislocation cells with the increase of strain (Fig. 2(f)). These results indicate that both Si content and grain size affect the dislocation evolution process significantly during tensile deformation, which will further influence the tensile stress-strain curves and strain-hardening behaviors. Therefore, in the following section, the two factors (dislocation slip mode (or SFE) and grain size) will be discussed to reveal the strain-hardening behavior of the Ni-Si alloys. Besides, the general mechanism for RSHR that can be found in several specific metals will be reviewed and revealed.

## Discussion

### The reason for the RSHR in Ni-4.5wt.%Si alloy

For Ni-4.5 wt.%Si alloy with a grain size of 200 μm, only one slip system can be observed at the beginning of plastic deformation because of its relatively low SFE, and the dislocations glide in planar mode, as shown in Fig. 2(a). In this case, the dislocation mean free path (Λ) at such a small strain should be very large (approximately equals to the diameter of grains if dislocations are emitted from grain boundaries). This may lead to a low density of mobile dislocations to coordinate the applied strain, which results a low Θ at the beginning of tension for Ni-4.5 wt.%Si alloy, as shown in Fig. 1(b,d). As deformation processes, the primary slip systems will be impeded so that the second slip system will be activated gradually, as shown in Fig. 2(b). In this case, the latter activated dislocations in the second slip system will be inhibited by the primary ones, shortening Λ of mobile dislocations sharply. And then, dislocation density must increase suddenly (as shown in Fig. 2(b)), leading to an accelerating strain hardening and the occurrence of RSHR for Ni-4.5 wt.%Si alloy. Therefore, the RSHR should originate from the indispensable event: **the plunging Λ caused sharp increase of dislocation density during deformation**, **which is facilitated by planar slip mode and large grain size**.

### The influence of slip mode on strain-hardening behavior

For the Ni-0.5 wt.%Si alloy with a grain size of 200 μm and the Ni-4.5 wt.%Si alloy with a grain size of 30 μm, the RSHR did not happen because of either wavy slip mode or small grain size. For the Ni-0.5 wt.%Si alloy, the dislocations will glide in wavy mode mainly because of its higher SFE^29^. And then, at the beginning of plastic deformation, the dislocations in Ni-0.5 wt.%Si alloy should be easy to entangle with each other because the cross slip tends to occur, leading to a small Λ^24^,^30^ and a high density of dislocations, as shown in Fig. 2(e). In this case, strain-hardening rate Θ of Ni-0.5 wt.%Si alloy in the initial stage of plastic deformation should be higher than that of Ni-4.5 wt.%Si alloy with the same grain size, as obviously indicated by Fig. 1(b). As the strain increases, microstructure evolution of Ni-0.5 wt.%Si alloy is characterized by the development of dislocation cells, as shown in Fig. 2(d). Nevertheless, this process will not involve the indispensable event for the Θ recovery, i.e. plunging Λ caused sharp increase in dislocation density, for the reason that the evolution of dislocation cells is a successive process and then the decrease of Λ is successive during plastic deformation (because Λ is mainly determined by the size of dislocation cells). Therefore, monotonous falling rather than the RSHR was found in Ni-0.5 wt.%Si alloy with a grain size of 200 μm.

Besides, in order to exclude the size effect, we have produced some Ni-0.5 wt.%Si samples with extremely large grain size (~1 mm) that can be observed by naked eyes. The strain-hardening rate of such samples does not experience a recovery. Therefore, the recovery of coarse grained Ni-4.5 wt.%Si and the absent recovery of coarse grained Ni-0.5 wt.%Si should be owing to the dislocation slip mode, rather than the size effect.

### The influence of grain size on strain-hardening behavior

Based on the mode of double pilling-up of dislocations, the number of piled-up dislocations (*n*) in one array with a pilling-up space *L* under a shear stress *τ* can be written as:


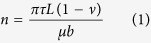


in which *ν* is the Poisson’s ratio, *μ* is shear modulus and *b* is the modulus of Burgers vector. At the very beginning of plastic deformation, dislocations rarely entangle with each other and can glide freely for a large distance. On the basis of an average assumption, the pilling-up space *L* can be the half of the grain diameter, 0.5*d*. Besides, based on the Orowan’s estimations on the strain relaxation after the sweeping over of a dislocation^31^, the spacing between dislocation arries can be expressed as:


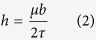


Thus the expression *hL* represents the average area occupied by one array of piled-up dislocations. In terms of the yield theory of polycrystals, when yield happens, dislocations may pile up at grain boundaries with a concentration stress 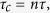 which should be approximately the same for microstructures with different grain size. Therefore, the effect of grain size on the dislocation density *ρ* at the yield point can be estimated as:


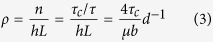


Such a reciprocal relationship between grain size and dislocation density was also obtain by a recent calculation study in Ni^32^. By the way, combining with the Taylor equation, 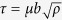, the famous Hall-Petch relation can be deduced. According to Eq. (3), with decreasing the grain size, the density of dislocations required for yielding increases, which reduces Λ at the beginning of plastic deformation. Besides, it needs higher density of mobile dislocations to coordinate a certain strain for smaller grains and stress states in smaller grains are more complicated so that second slip systems can be easily activated in small grains even at very low strain, as shown in Fig. 2(c). Just for these reasons, Θ of Ni-4.5wt.%Si alloy with a small grain size of 30 μm should be higher than that with a large grain size of 200 μm at the beginning of plastic deformation. However, Θ decreases quickly to zero for Ni-4.5wt.%Si alloy with grain size of 30 μm because of its low strain-hardening capacity, as shown in Fig. 1(d). In this case, Λ does not experience a sudden change during plastic deformation so that Θ recovery does not happen for Ni-4.5 wt.%Si alloy with small grains. Therefore, although the dislocations in Ni-4. 5wt.%Si samples with different grain sizes glide in the same mode, i.e. planar slip mode, the difference is that the smaller the grain size is, the more are the dislocation systems required at the same strain level, as described in the revised manuscript.

In fact, besides these two grain sizes, we also prepared a series of polycrystalline samples with grain sizes between 30 μm and 200 μm for Ni-4.5wt.%Si alloy through changing the annealing temperature, and the change of Θ with strain of these samples accords with the discussions and conclusions above. It may be estimated that the critical grain size for Θ recovery in Ni-4.5 wt.%Si should be between 160 ~ 190 μm.

**Figure 3:**
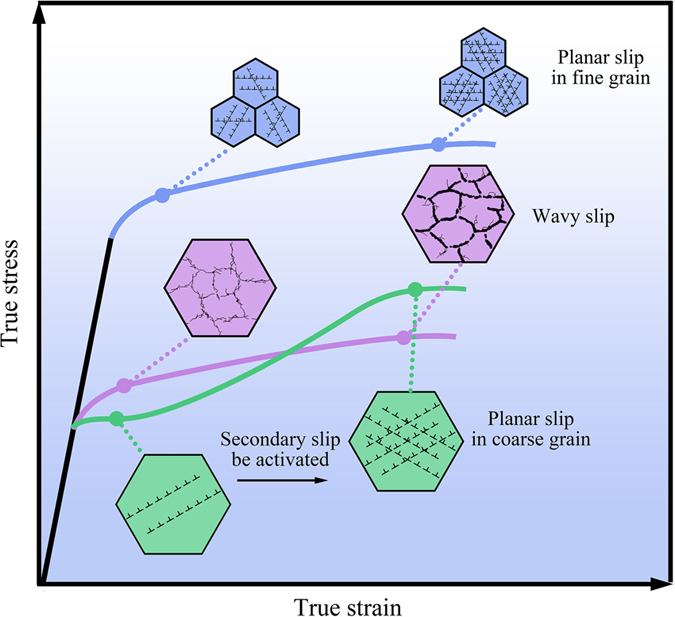
Schematic illustration on the strain-stress curves and dislocation evolution during tension for the three typical materials with dislocation activities alone, planar-slip metals with large/small grain sizes and wavy-slip metals. The RSHR effect can only arise in planar-slip metals with large grain size.

On account of the analyses and discussions above, Fig. 3 schematically illustrates the tensile stress-strain curves and microstructure evolution during deformation for the three typical materials: planar-slip materials with coarse and fine grains and wavy-slip materials, from which the effect of slip mode and grain size on dislocation structure evolution and tensile stress-strain curves (or Θ change) can be clearly indicated. The only stress-strain curve with Θ recovery is the one with planar-slip dislocation mode and relatively large grains, mainly because of the multiplication of secondary slip systems during deformation process.

### The general mechanism for RSHR during tension

**Figure 4:**
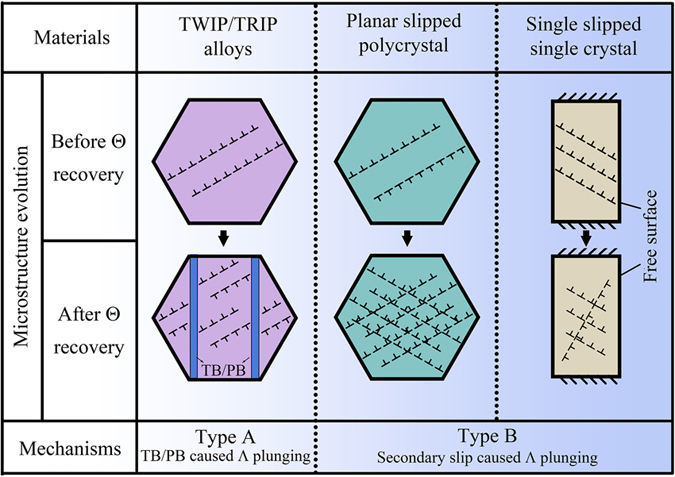
Summary and category of materials that possess the RSHR effect. RSHR in different materials shares the same intrinsic mechanism, the sharp decrease of dislocation free path (Λ) during tension caused either by planar defects (for TWIP/TRIP alloys) or by dislocations (for planar-slip coarse grain or single-slip single crystal).

Considering the other three kinds of materials that exhibit the RSHR phenomenon mentioned in the introduction, the TWIP alloys (including those only produce stacking faults), TRIP alloys and single-slip single crystals, the general mechanism for the RSHR of these materials should be the same as that in the Ni-4.5 wt.%Si alloy with coarse grain in this study, i.e. **plunging Λ caused sharp increase of dislocation density during deformation**. Figure 4 concludes the concrete mechanisms for Λ plunging of these materials, in which two categories can be divided: type A (boundary caused Λ plunging and Θ recovery) and type B (dislocation caused Λ plunging and Θ recovery). For the TWIP and TRIP alloys, they can be classified into the type A because the Λ plunging is the newly formed boundaries (twin boundaries (TBs) or phase boundaries (PBs)) during plastic deformation. However, the materials with dislocations in a planar-slip mode and large grain discovered by the present study can be classified into the “type B”, as its Λ plunging is caused by an activated secondary slip system. For single-slip single crystals that have work-hardening stages I and II, the obvious Θ recovery from stage I to II can be contributed to two reasons: (a) secondary slip system caused Λ plunging; and (b) dislocation interaction caused inhibition to the dislocation annihilation at free surfaces. Both of these reasons derive from the activated secondary slip dislocations, so it can also be classified into type B. The recovery of Θ in single crystal only appears for single-slip orientations. However, for polycrystals, single slip cannot happen to appear in all grains. So, the mechanisms of Θ recovery in single crystal and polycrystals are not the same.

To sum up, the work-hardening behaviors and microstructure evolution of two Ni-Si alloys with two different grain sizes were investigated by axial tensile tests. The RSHR was first discovered in polycrystalline materials (Ni-4.5 wt.%Si alloy) that only activated dislocations during plastic deformation. Further observations confirmed that the RSHR may only arise in the polycrystals with large grain size and planar slip mode. After the theoretical analysis, the general microscopic mechanism for the appearance of RSHR was revealed, i.e. **plunging** Λ **caused sharp increase of dislocation density during plastic deformation**. The absence of such a recovery in the Ni-0.5wt.%Si alloys should be the result of the mobile dislocations with wavy slip mode, which facilitates dislocation annihilation. As to the Ni-4.5 wt.%Si alloy with fine grain, the restriction of grain boundaries on dislocation motion may trigger the activation of secondary slip systems at the very beginning of plastic deformation, which also leads to the absence of RSHR. All the materials that possess the RSHR are further summarized and classified into two types based on their concrete mechanism for the plunging Λ.

## Methods

### Materials fabrication

On account of the phase diagram, the solubility of Si atom in Ni lattice is as much as 5 wt.% at room temperature. Therefore, two Ni-Si alloys with relatively low Si contents were selected, Ni-0.5 wt.%Si and Ni-4.5 wt.%Si, to insure the absence of second phase. Meanwhile, the SFE values of Ni-0.5 wt.%Si and Ni-4.5 wt.%Si alloys are 151.5 mJ/m^2^ and 90.6 mJ/m^2^, respectively, which were calculated by means of the full potential linearly augmented plane wave incorporating local orbital method based density functional theory (DFT), and the detailed computation course was presented elsewhere^33^. All the cast ingots were kept at 1423 K for 2 hours, then immediately hot forged to square rods with final dimension of 25 × 25 mm^2^ at 1123 ~ 1423 K, and finally cooled in air. After that, these rods were hot rolled at 400°C, and then annealed at two different temperatures, 450 °C and 1000 °C, for 90 minutes to obtain different grain sizes of about 30 and 200 μm, respectively. The specimens were sparking cut along the axial direction with gauge sectional dimensions of 15 × 4 × 3 mm^3^.

### Tensile test

Tensile tests were carried out at a strain rate of 10^−3^s^−1^ in an INSTRON 5982 testing machine at room temperature. And for each case we conducted three experiments, which ensures the repeatability and credibility of the results.

### Microstructure characterization

No secondary phase, precipitation or element concentration was detected before and after tensile tests by X-ray diffraction (XRD), SEM-EDS and STEM-EDS element mapping. TEM and XRD patterns indicate that the lattice structures of these alloys are FCC with a similar lattice constant (~0.353 nm). Therefore, the solution of Si atoms does not affect the lattice of Ni severely. In order to study the microstructural evolution, interrupted tensile tests at true strain of 0.02 and 0.12 were performed. The microstructures of the deformed samples were investigated with an FEI Tecnai F20 transmission electron microscope (TEM) operated at 200 kV. All the TEM photos were taken under two-beam condition with g = (111), nearby the (−110) pole.

## Additional Information

**How to cite this article**: Yang, C. L. *et al.* Recovery of strain-hardening rate in Ni-Si alloys. *Sci. Rep.* **5**, 15532; doi: 10.1038/srep15532 (2015).
